# A physical sign of pathological myopia: myopic scleral pit

**DOI:** 10.1186/s12886-023-02847-y

**Published:** 2023-03-22

**Authors:** Wenhua Zhang, Yaping Zhang, Jingxuan Xu, Handong Dan, Xiaoli Li, Zongming Song

**Affiliations:** 1 Henan Provincial People’s Hospital, Henan Eye Hospital, Henan Eye Institute, Zhengzhou, 450003 Henan China; 2 Shaanxi Eye Hospital, Xi’an People’s Hospital (Xi’an Fourth Hospital), Affiliated People’s Hospital of Northwest University, Xi’an 710004, China; 3 Zhengzhou University People’s Hospital, Henan Provincial People’s Hospital, Henan Eye Hospital, Zhengzhou, 450003 Henan China

**Keywords:** Pathological myopia, Myopic scleral pit, Physical sign

## Abstract

**Purpose:**

Myopic scleral pit (MSP) is a rare physical sign of pathological myopia (PM). The aim of this study was to summarize the clinical characteristics of MSP and analyze its correlation with PM.

**Methods:**

Eight cases with PM and MSP were enrolled in this study. Comprehensive ophthalmic examinations, including subjective refraction, slit-lamp biomicroscope, intraocular pressure, fundus photographs, A- and B-scan ultrasonography and spectral-domain optical coherence tomography, were performed.

**Results:**

All the patients had a long history of PM with visual impairment, long axial length, and myopia-related fundus degeneration. Mean axial length was 31.48 ± 2.17 mm. Mean size of MSP was 0.69 ± 0.29 optic disc diameter (PD). Mean logMAR BCVA was 1.21 ± 0.88 logMAR. Spearman correlation analysis showed that the logMAR BCVA had no correlation with the size of pits (*P* = 0.34). Fundus examination revealed a focal pale concave located in the sclera exposed area of retinal choroid atrophy was found in all cases. OCT showed a deep scleral pit where the retinal choroid was thin or absent, without retinal sensory detachment or sensory defect.

**Conclusions:**

This study identified a rare scleral lesion in all eight individuals with PM, which was termed “myopic scleral pit”. This phenomenon is different from focal choroidal excavation and posterior staphyloma.

## Introduction

Pathological myopia (PM) is a common ophthalmopathy and a major cause of blindness worldwide [1]. Holden et al. predicted that by 2050, approximately 50% of people would suffer from myopia, and about 10% will have high myopia [2]. Thus, myopia has become a major global concern. PM is defined as myopia accompanied by characteristic myopic fundus changes. The presence of myopic maculopathy is equivalent to or more serious than diffuse choroidal atrophy or the presence of posterior staphyloma [3]. Myopia-related fundus changes include posterior staphyloma, myopic maculopathy, myopic traction maculopathy, and optic disc changes [4]. The main complication of myopia is myopic macular degeneration (MMD), which is a common cause of visual impairment, especially for high myopia [5]. It is characterized by lacquer cracks, Fuchs spot, choroidal neovascularization, or chorioretinal atrophy [6]. Posterior staphyloma is a specific type or a risk factor for the development of MMD [7]. Retinal detachment, pigmentary degeneration, lattice degeneration, and paving stone degeneration are common peripheral retinopathies in patients with high myopia; among these, retinal detachment poses a maximum threat to vision [8].

Furthermore, little is known about the pathogenesis of myopic scleral pit (MSP). In the previous two cases [9, 10], this phenomenon has been described as macular pit and myopic macular pit, respectively. These pits showed a sudden change in the scleral curvature from the surrounding area, which could be single or multiple. Notably, macular pits occurred in the area of chorioretinal macular atrophy, illustrating that the continuous mechanical tension onto chorioretinal macular atrophy leads to MSP [9].

The clinical characteristics of eight patients diagnosed with PM combined with MSP were analyzed in this retrospective study. An oval pit was found below the macula lutea in all cases. Thus, MSP may be a rare physical sign of PM or congenital anomalies of the sclera.

## Patients and methods

Patients aged 12–67 years with unilateral or bilateral PM at the Henan Provincial People’s Hospital and the Eye Hospital of Wenzhou Medical University from 2008 to 2020 have been reviewed. According to the inclusion and exclusion criteria, eight patients (eight eyes) with ophthalmic examination results were enrolled in this study. Visual impairment and myopia-related fundus changes were detected in all patients.

### Related inspection and devices

Each eye was performed a comprehensive ophthalmic examination, including the best-corrected visual acuity (BCVA), slit-lamp biomicroscope, intraocular pressure (Tx-20 automatic non-contact tonometer, Shanghai Langyi Medical Instrument LTD, CANON, Japan), fundus photographs, A- and B-scan ultrasonography (MD-2400S, Tianjin Mida Medical Technology Ltd, Tianjin, China) and spectral-domain optical coherence tomography (SD-OCT) (Spectralis OCT, Heidelberg Engineering, Heidelberg, Germany). 

### Visual acuity representation method

The visual acuity was calculated as follows: V = d/D, where V is the visual acuity, d is the actual distance of seeing a test object, and D is the distance that normal eyes should be able to see the test object. The patient was examined based on an international standard visual acuity chart, with normal far-visual acuity of 1.0. If the maximum visual beacon cannot be identified at 5 m (0.1 row), the subject was instructed to step towards the visual acuity chart until the visual beacon could be identified. If the maximum visual index cannot be identified at 1 m on the visual acuity table, the counting fingers (CF), hand motions (HM), light perception, or no light perception should be checked. The test method of CF was as follows: the examiner held out different numbers of fingers and asked the subject to indicate the number of fingers. The inspection distance started from 1 m and gradually moved closer until it could be identified correctly, and the distance was recorded. BCVA was recorded as the logarithm of minimal angle of resolution ( LogMAR), with counting fingers reaching 2.3 logMAR [11].

The inclusion criteria for the clinical cases were as follows: One or more well-defined oval pits were observed in the fundus of PM, with thinning and outpouching of the retina, retinal pigment epithelium (RPE), choroid, and sclera. The neurosensory retina and RPE in the pit became thinner, and the closer it was to the center of the depressed area, the thinner it was, but no retinal detachment was observed. Such scleral depression was diagnosed as MSP and included in this study. The included patients were > 18-years-old and had mature ocular, which could be combined with other myopia-related fundus lesions. Patients with ocular trauma, posterior segment surgery, and incomplete clinical data were excluded.

### Statistical analysis

SPSS 21.0 software was used for all statistical analyses. Levene’s test was used to test for homogeneity of variance, and the Shapiro–Wilk test was used to assess data normality. Data were expressed as mean values and standard deviations (SD). Spearman correlation analysis was used to evaluate the correlation between BCVA and the size of pits. *P*-values less than 0.05 were considered statistically significant.

## Results

Eight eyes (left, *n* = 3; right, *n* = 5) from eight patients (three men and five women) were analyzed in this retrospective study. The mean age of patients at the time of definite diagnosis was 59.88 ± 5.59 years (range, 54–66 years). The enrolled patients had high myopia for > 30 years with severe myopic retinal degeneration. Visual acuity impairment, axial elongation in eyes, leopard fundus, diffuse chorioretinal atrophy, pigment clumping, choroidal neovascularization, myopic conus and posterior staphyloma were observed in all eyes (100%). Retinal lattice degeneration and refractive medium opacity were detected in four eyes (50%). Lens dislocation and epiretinal membrane were diagnosed in three eyes (37.5%). Retinoschisis was found in one eye (12.5%).

**Fig. 1:**
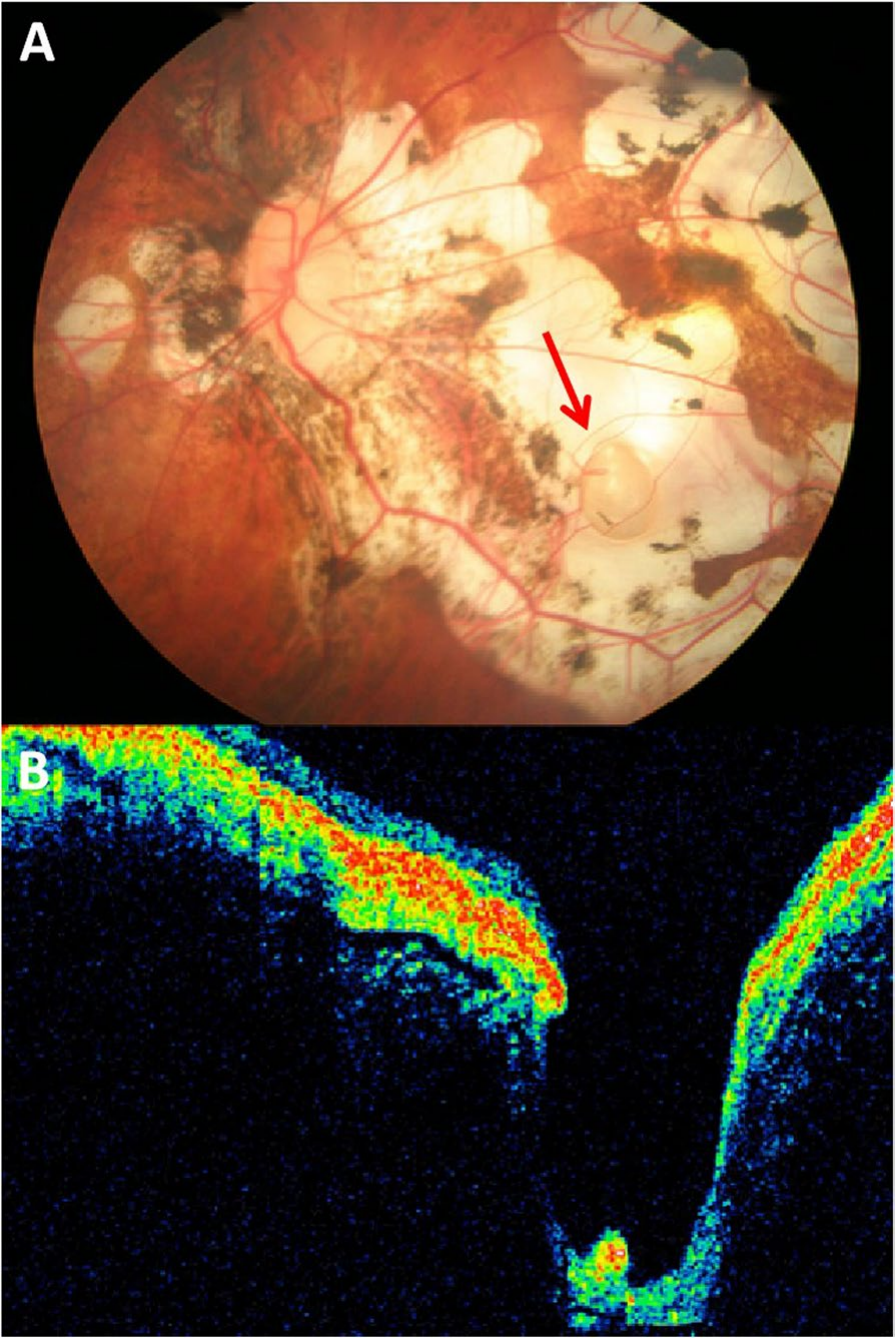
Left eye of a 62-year-old man with pathological myopia and metamorphopsia (Case 1). **A** Fundus photograph: The eye had severe myopic retinal degeneration, such as atrophy of the choriocapillaris, choroid lacquer cracks, and retinal thinning. A well demarcated oval gray white lesion with linear retinal vessels at the base was visible inferotemporally. The pit was approximately one optic disc diameter. **B** OCT: A crater-like and oval-shaped depression was visible on the temporal side, approximately 30 μm from the fovea centralis. Hyperintensity was detected below the neurosensory retina, with an unknown correlation to the RPE. No neurosensory retinal detachment or neurosensory defect was detected

**Fig. 2:**
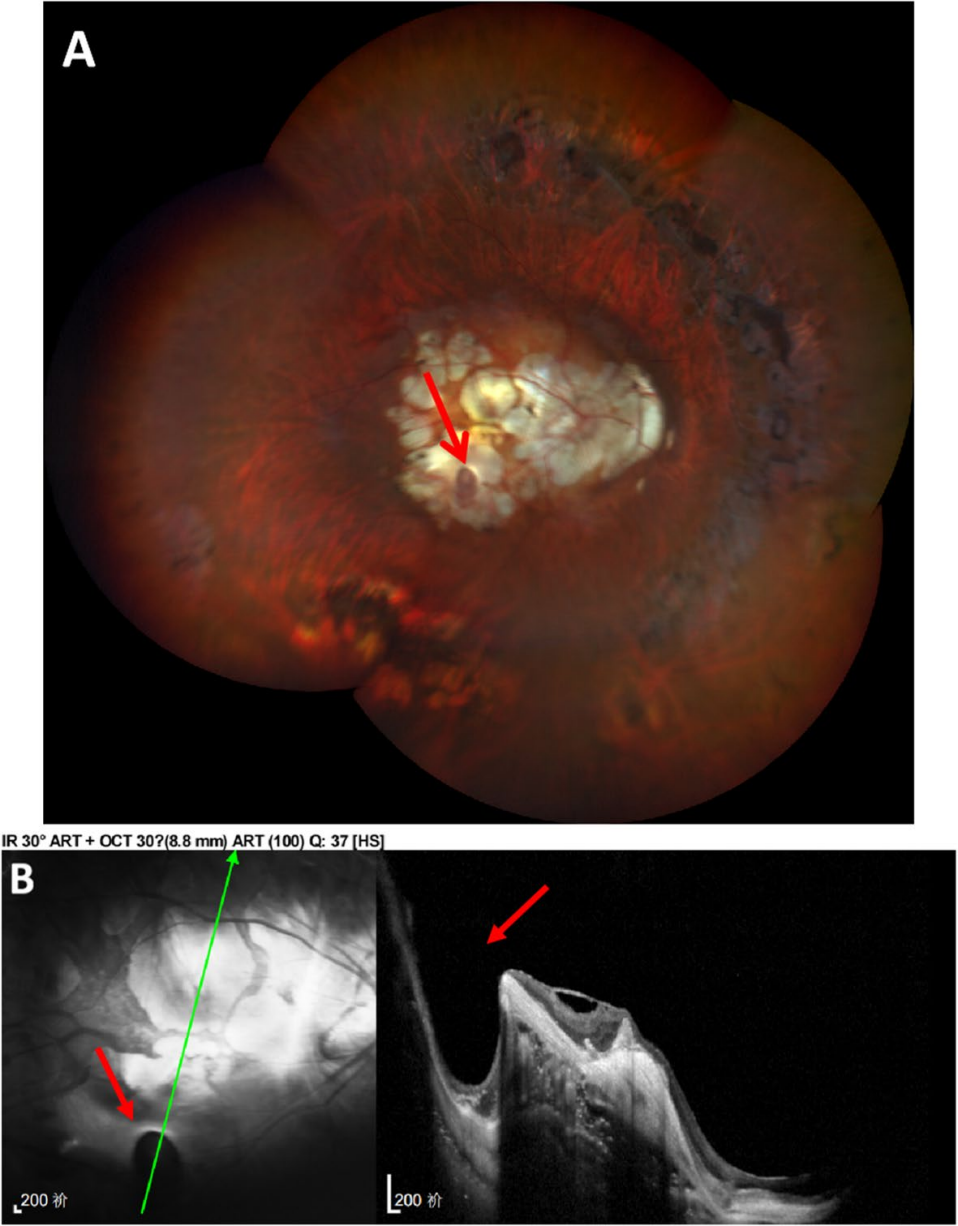
Right eye of a 66-year-old woman with pathological myopia and dislocation of the lens (Case 4). **A** Fundus photograph: Severe retinal and choroidal atrophy with sclera exposure was seen in the posterior pole. Myopia conus was observed in the temporal side of the optic disc. Large-area degeneration and focal involvement were detected around the whole retina. Below the macular area, there was a well-defined oval depression with a size of about two-thirds PD. Lateral branches of retinal vessels at the bottom of the depression. **B** OCT: The pit was 584 μm in depth and 1283 μm in width. Hyperintense reflects were seen below the neurosensory retina, with an unknown correlation to the retinal pigment epithelium. A macular epiretinal membrane was formed, resulting in retinal traction. Some retinal and choroidal tissue defects with thin choroidal and posterior scleral staphyloma. The central retinal thickness was 76 μm

**Fig. 3:**
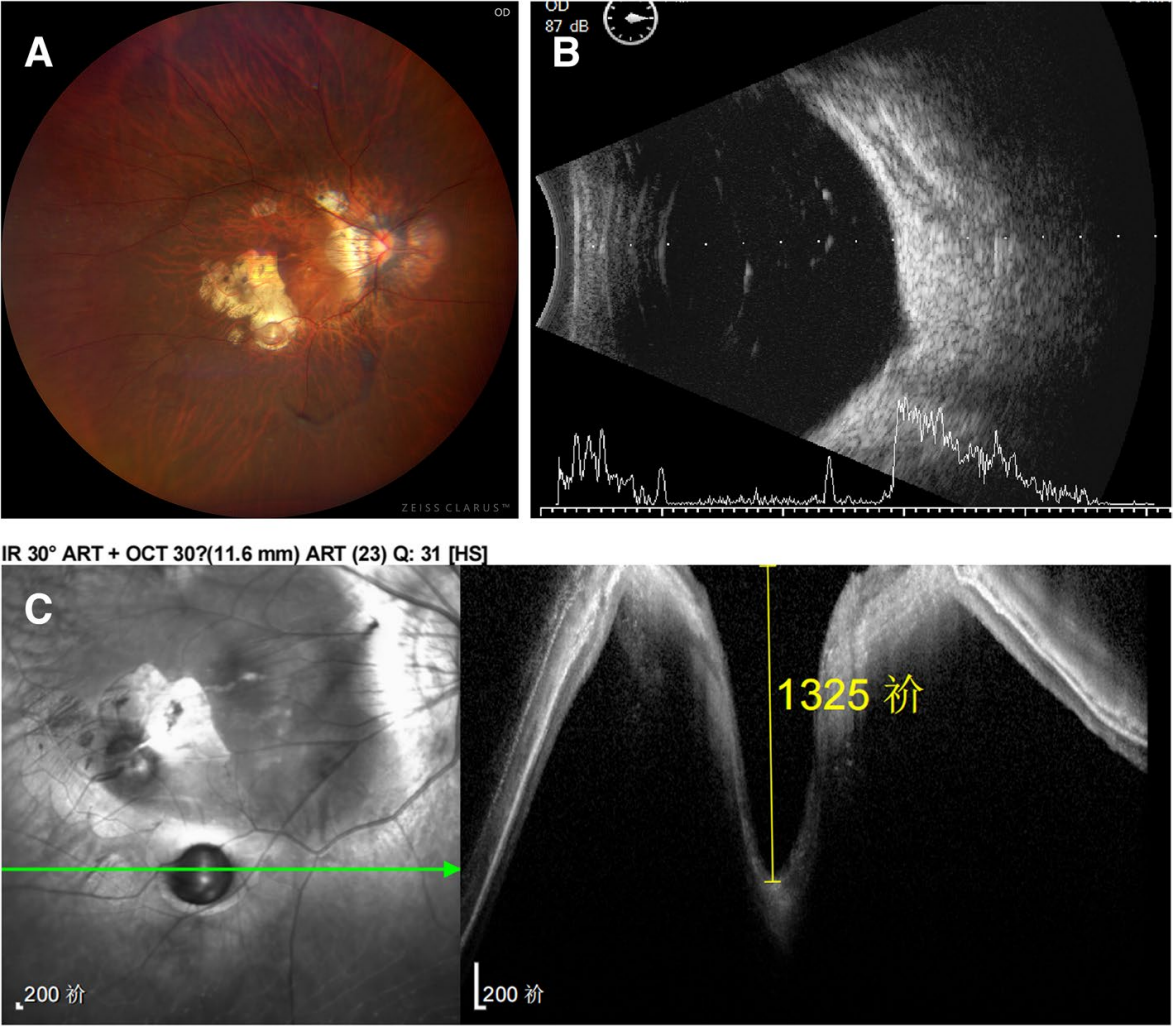
Right eye of a 56-year-old woman with pathological myopia and metamorphopsia (Case 6). **A** Fundus photograph: Fundus changes in high myopia. A well-defined grayish oval depression was located in the sclera exposed area. **B** A- and B-scan ultrasonography showed vitreous opacity and posterior vitreous detachment. A small depression was seen on the ocular wall. **C** OCT revealed a deep pit-like depression in the atrophy area, with a depth of more than 1325 μm

**Fig. 4:**
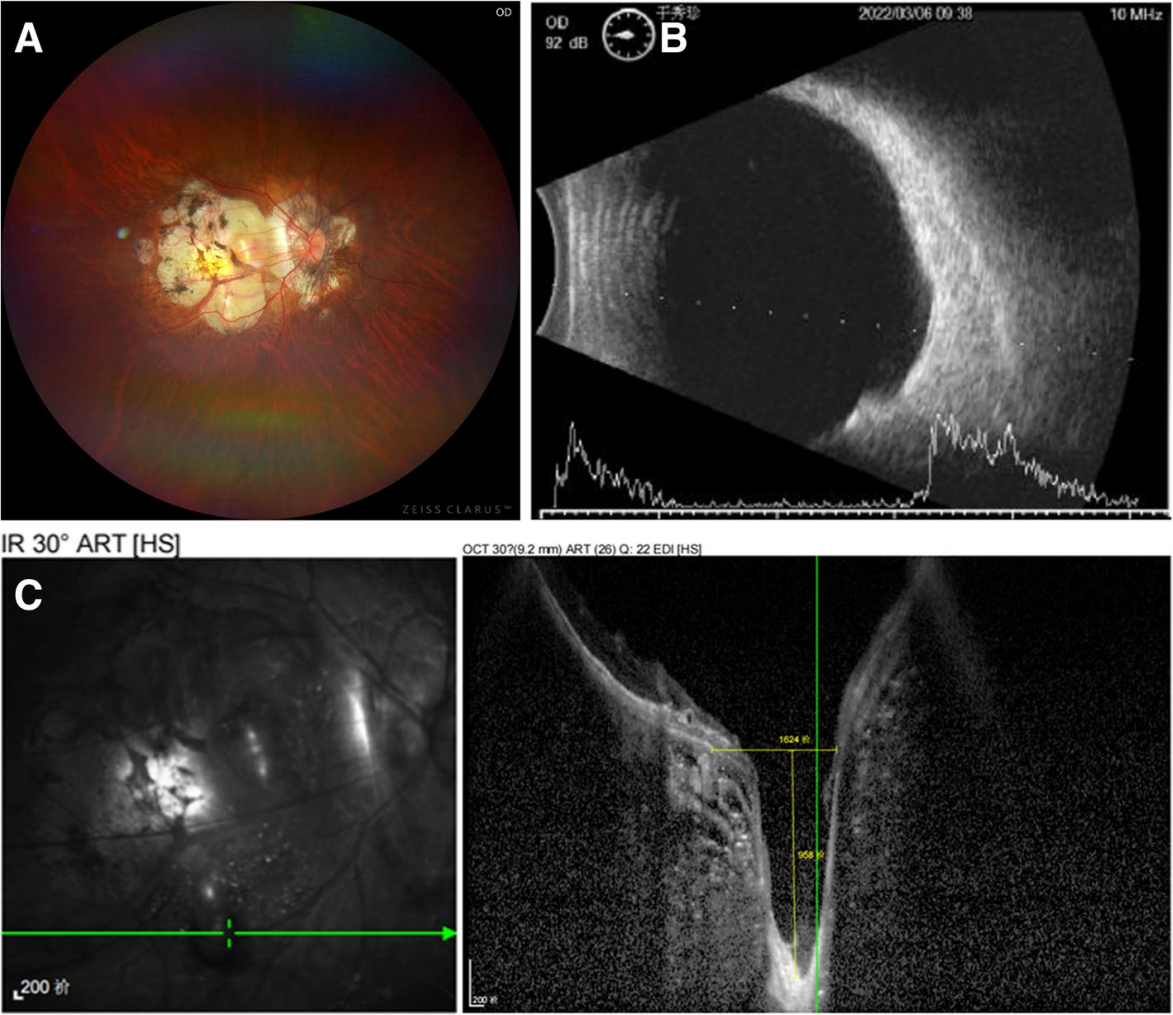
Right eye of a 57-year-old woman with pathological myopia ( Case 7).** A** Fundus photograph: The posterior pole of retina and choroid was atrophic. A grayish oval depression could be seen below the macula. **B** A- and B-scan ultrasonography showed the significant posterior staphyloma. **C** OCT demonstrated a deep pit with a depth of 958 μm

**Fig. 5:**
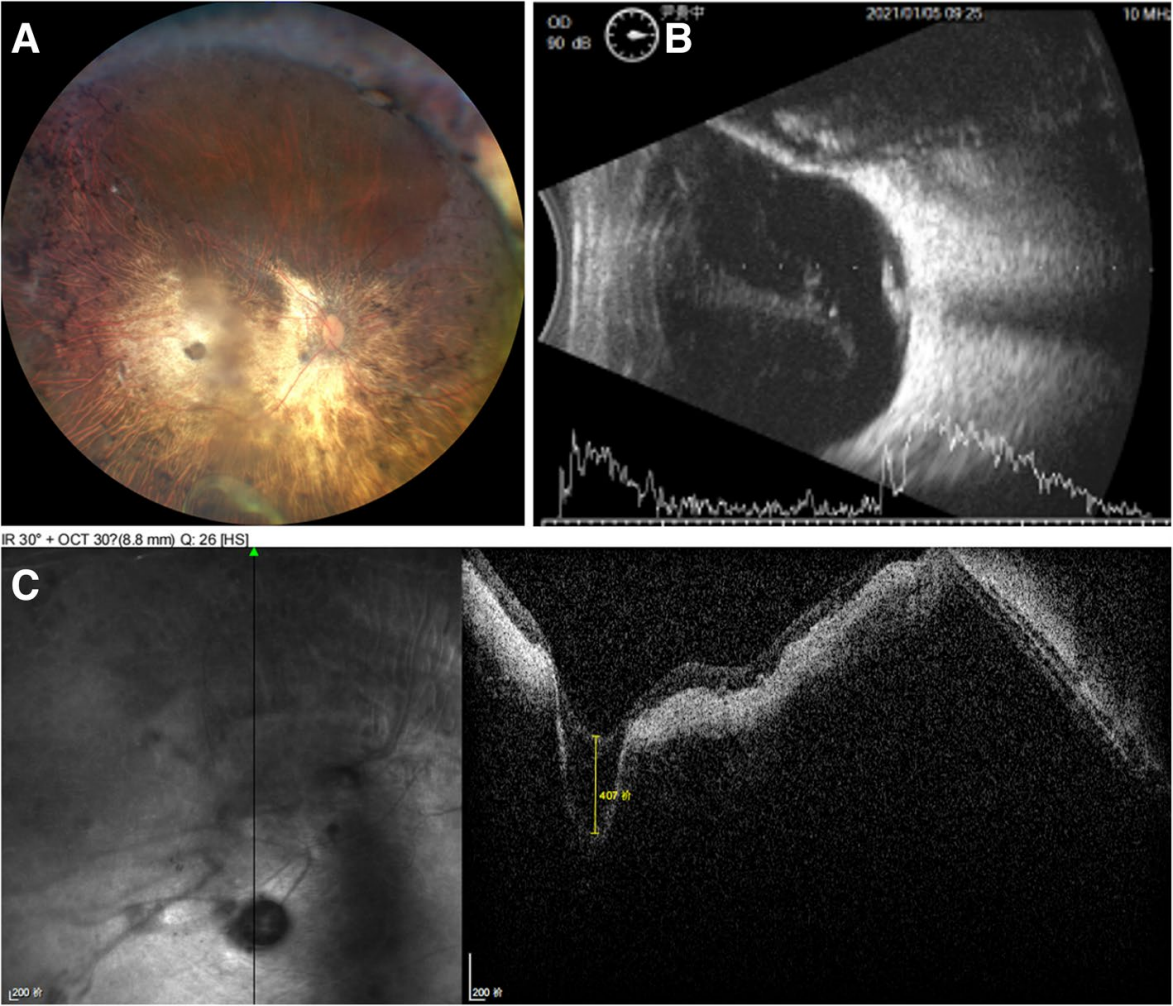
Right eye of a 70-year-old man with pathological myopia ( Case 8). **A** Fundus photograph showed extensive retinal thinning and absence of choroid. A round gray pit could be observed below the macula. **B** A- and B-scan ultrasonography showed the refractive medium opacity and lens dislocation. **C** OCT demonstrated the pit with a depth of 407 μm

The fundus photo showed severe myopic retinal degeneration in all cases. The deep choroidal atrophy in the posterior pole exposed the sclera. A pit near the macula lutea could be observed in the region of retinochoroidal atrophy. OCT revealed the deep pit with thinning or even partial loss of retina and choroid (Figs. 1, 2, 3, 4 and 5). The sclera was exposed in the pit, which was described as an MSP in this study. BCVA ranged from 0.2 to 2.3 logMAR, with an average of 1.21 ± 0.88 logMAR. MSPs were located below macula lutea (75%, *n* = 6) and nasal inferior macula lutea (25%, *n* = 2). The axial lengths ranged from 28.56 mm to 35.15 mm. The mean axial length was 31.48 ± 2.17 mm. The size of MSPs ranged from 0.5 Optic disc diameter (PD) to 1 PD, with an average of 0.69 ± 0.29 PD. The clinical characteristics of the patients and pits are listed in Table 1. Spearman correlation analysis showed that the logMAR BCVA had no correlation with the size of pits (*P* = 0.34) (Table 2).

**Table 1:** Summary of patients’ clinical characteristics

Case No	Sex	Age (years)	Eye	Axial length (mm)	BCVA (logMAR)	OCT	Pit Size (PD)	Location	Myopia-related fundus changes	Case resource
1	M	62	L	35.15	0.4	Hyperintense reflects were seen below the neurosensory retina, with an unknown correlation to the RPE. There was no neurosensory retinal detachment or neurosensory defect	1	Below macula lutea	LF, CRA, PC, CNV, PS, MC	This study
2	F	54	R	28.56	0.2	The pit was approximately 776 μm × 457 μm × 330 μm in size. There was no neurosensory retinal detachment or neurosensory defect	1/3	Below macula lutea	LF, CRA, PC, CNV, PS, MC, RLD	
3	F	57	L	30.10	1.7	The neurosensory retina and RPE became thinner. No retinal neuroepithelium detachment and loss were found. The depression depth was 1518 μm	1	Nasal inferior macula lutea	LF, CRA, PC, CNV, PS, MC, RLD	
4	F	66	R	32.29	2.3	ERM resulted in retinal traction. Retinal and choroidal tissue defects and thinning. The pit was 584 μm in depth and 1283 μm in width	2/3	Below macula lutea	LF, CRA, PC, CNV, PS, MC, RLD, LDL	
5	M	57	L	31.83	2.3	The neurosensory retina in the pit became thinner, and the closer it was to the center of the depressed area, the thinner it was, but there was no detachment or loss of retinal neuroepithelium	1	Nasal inferior macula lutea	LF, CRA, PC, CNV, PS, MC, LDL, ERM	
6	F	56	R	31.73	0.7	The fovea was thickened and raised, pulled by the anterior membrane, with a thickness of 300 μm. Cystoid edema could be observed. Choroidal atrophy, thinning and even missing. The depth of the scleral depression was greater than 1325 μm	2/3	Below macula lutea	LF, CRA, PC, PS, MC, ERM	
7	F	57	R	33.10	0.4	The anterior retina, retinoschisis, partial loss of retinal pigment epithelium, and posterior scleral staphyloma were found in the macular region and posterior pole of the retina, while the retina, choroid, and sclera at the pit were thin. The depth of the scleral pit was 958 μm	1/2	Below macula lutea	LF, CRA, PC, PS, MC, ERM, R	
8	M	70	R	29.10	1.7	The posterior pole of retina was thinned with irregular RPE. The choroid was atrophied and thinned with a significant depression. The depth of the depression was 407 μm	1/3	Below macula lutea	LF, CRA, PC, PS, MC, RLD, LDL	
9	F	73	R	32.00	1.1	Pits with a sudden change of scleral curvature from the surrounding area were observed. The hyperreflective scleral tissue was observed at the bottom of the pit. The neural retina was observed on the hyperreflective scleral tissue at the bottom of the pit, and it seemed to be continuous from the retina around the pit. The depth of these three pits from their openings was 584 μm, 715 μm, and 444 μm	/	Macular region/ Nasal inferior macula/ Superior temporal macular	/	Case of Ohno-Matsui et al
10	F	57	L	/	1.5	Loss of retina, RPE, choroid, and sclera could be observed. The pit was inferior to the fovea with 902 μm in depth and 397 μm in width	1/5	Below macula lutea	/	Case of Vadivelu J et al

*M* Male, *F* Female, *R* Right, *L* Left, *RPE* Retinal pigment epithelium, *LF* Leopard fundus, *CRA* Chorioretinal atrophy, *PC* Pigment clumping, *CNV* Choroidal neovascularization, *PS* Posterior staphyloma, *MC* Myopic conus, *RLD* Retinal lattice degeneration, *LDL* Lens dislocation, *ERM* Epiretinal membrane, *R* Retinoschisis

**Table 2:** Spearman correlation analysis of BCVA and size of pits

		Size of pits (PD)
**BCVA (LogMAR)**	Spearman r	0.39
	*P*	0.34

*BCVA* Best corrected visual acuity, *LogMAR* Logarithm of minimal angle of resolution, *PD* Optic disc diameter

The first patient that we diagnosed was a 62-year-old man with a history of PM for 50 years (case 1). He had undergone intraocular lens implantation in both eyes for ten years and the uncorrected vision was not improved. His BCVA was 0.3 logMAR with -2.00DS/-1.25DC × 148 in right eye and 0.4 logMAR with -2.00DS/-2.00DC × 150 in left eye. The axial length of the two eyes was 34.47 mm and 35.15 mm. His left eye showed a sharply-demarcated, oval-shaped, excavated lesion 4 mm temporal to the optic disc center with lateral branches of the retinal vessels at the base. The pit was approximately one PD (Fig. 1A). OCT revealed the crater-like depression about 30 μm temporal to the central macular fovea and at the infratemporal edge of the fovea (Fig. 1B). A 66-year-old female presented with vision decreased for more than 4 months in the right eye after cataract surgery for 6 years, who had a history of PM for more than 40 years (case 4). The BCVA was 2.3 logMAR in the right eye and 0.7 logMAR with -9.50DS/-1.00DC × 60 in the left eye. Axial length was 32.29 mm (right) and 32.90 mm (left). Intraocular lens of her right eye was dislocated downward into the vitreous cavity. The fundus examination of both eyes showed atrophy arc around the optic disc. Retinal choroidal atrophy could be seen at the posterior pole, where the sclera was exposed. Significantly, in her right eye, a well demarcated oval shaped depression approximately two-thirds of PD in size was found in the scleral exposure area (Fig. 2A). OCT showed heterogeneous light clumps beneath the macular neuroepithelium with unclear relationship to RPE. A small amount of anterior macular membrane was formed, resulting in retinal traction. Some retinal and choroidal tissue defects with focal downward depression, thin choroidal and posterior scleral staphyloma. The central retinal thickness was 76 μm (Fig. 2B). Case 6 was a 56-year-old female who presented with over 40 years of reduced visual acuity and a 2-year metamorphopsia in her right eye. The BCVA of both eyes was 0.7 logMAR (right) and 0.2 logMAR (left). The axial length of the right eye was 31.73 mm and the left eye was 28.70 mm. Retinal choroidal atrophy, myopic arc, severe leopard-shaped fundus could be observed in her right eye. A well-defined grayish oval depression was located in the sclera exposed area (Fig. 3A). A- and B-scan ultrasonography showed vitreous opacity and posterior vitreous detachment. A small depression was seen on the ocular wall (Fig. 3B). OCT revealed a deep pit-like depression in the atrophy area, with a depth of more than 1325 μm (Fig. 3C).

## Discussion

The MSP shown in patients with high myopia is characterized by a deep pit near the macular area, with abnormal overlying retinal architecture, thinning, and outpouching of the retina, RPE, choroid, and sclera. There was no retinal detachment or traction in the lesion area, but discontinuity or absence could be observed in retina. The continuous growth of the axial length in pathological myopia patients could cause choroidal ischemia hypoxia, which in turn leaded to retinal and choroidal atrophy. Eventually, the effect of intraocular pressure may contributed to the form of pits. These findings provide us a new phenotype of anomalies of the sclera, which might be a scarce physical sign of PM.

Such manifestation is not yet widely reported in the literature. The appearance of macular depression and optic disc fovea is similar; however, in macular fovea, scleral tissue exists at the bottom of the fovea and is continuous with the surrounding sclera [3]. Ohno-Matsui et al. described three pits of one eye. Fundus photographs showed that the pits in the patient with pathological myopia were developed within the area of chorioretinal macular atrophy. Also, pits with a sudden change in scleral curvature from the surrounding area were observed. The hyperreflective scleral tissue was seen at the bottom of the pit. The neural retina could be observed on the hyperreflective scleral tissue at the bottom of the pit, which was continuous from the retina around the pit. The depth of these pits from their openings was 584 μm, 715 μm, and 444 μm, respectively [9]. Vadivelu et al. considered that multimodal imaging was helpful for the evaluation of MSP. The study reported one case with detached retina and loss of RPE, choroid, and sclera, as assessed by swept-source OCT. The pit was lower than the fovea with 902 μm in depth and 397 μm in width. Also, episcleral tissue can be imaged in a few areas [10]. As described in our cases, these pits were located below the macular area and in the chorioretinal atrophy in high myopia. In these areas, RPE and choriocapillaris were absent and the underlying Bruch’s membrane and photoreceptors were disrupted. Despite the uniform distribution of intraocular pressure, the expansion of the posterior wall is pronounced in the areas of chorioretinal atrophy since the absence of both retinal and choroidal layers makes this area more vulnerable to pressure than normal areas [9]. During both retrobulbar anesthesia and vitrectomy or when any ophthalmic surgery was performed, the macular pit has the potential for serious complications or even scleral perforation due to intraocular pressure fluctuation, which should be observed cautiously.

MSP is different from focal choroidal excavation (FCE) and posterior staphyloma (Table 3). FCE is characterized by good visual sensitivity and nearly normal overlying retinal structure, which changed only slightly. Shinojima et al. classified the FCE into three morphological patterns based on OCT appearance as cone-shaped, bowl-shaped, or mixed-type [12]. The pathogenesis of choroidal depression is not clear [13, 14]. Idiopathic FCE may be associated with inflammatory diseases [15]. The “abnormal hyperreflective tissue” found by OCT below some FCE lesions may represent the scar of choroidal connective tissue during previous inflammation [16]. Thus, it is speculated that scar contraction can attract choroid and RPE to sclera-producing FCE, but focal choroidal atrophy is caused by subclinical choroidal inflammation or choroidal ischemia might lead to FCE formation without scar [17].

**Table 3:** Differential diagnosis of myopic scleral pit, focal choroidal excavation and posterior staphyloma

	**Myopic scleral pit**	**Focal choroidal excavation**	**Posterior staphyloma**
Location	Area of retinal choroid atrophy	Choroid without obvious posterior staphyloma or scleral dilation	Fundus posterior pole
OCT performance	"Pit-like changes" in which the retina and choroid are noticeably thinner or absent	The structure of the overlying retina is almost normal	The outer retina, RPE, choroid and sclera were thinning and outpouching with intact inner retina
Formation	Long-term pathological myopia	Moderate myopia with good visual sensitivity, mild blurred vision or deformation or no symptoms	Long-term pathological myopia
Fundus performance	Grayish—white oval depressions in atrophic areas	Normal or non-specific pigmentation, or blurred yellow-white spots in the lesion location	Clear dark brown rounded depression with hyperpigmentation above

Posterior staphyloma is an outpouching of the posterior shell of the eye frequently found in highly myopic eyes and is considered a hallmark of PM [18]. It is histologically characterized by abrupt scleral thinning beginning at the edge of the staphyloma. The distinct arrangement of scleral collagen fibers and pronounced choroidal thinning were obvious at the edge of the staphyloma [19]. Importantly, the formation of staphyloma is an independent phenomenon that develops from both the continuous extension of axial length and the eyes with non-axially elongation [20]. Subsequently, posterior staphyloma deepens, and its morphological features also change with increasing age [21, 22]. Posterior staphyloma is divided into five types: wide macular staphyloma, narrow macular staphyloma, peripapillary staphyloma, nasal staphyloma, and inferior staphyloma [23]. The scleral manifestations mentioned in this study are not included in the above classification. The MSP is located below the macula and its size is about one PD. OCT shows that the depth of MSP is greater than the width, forming a canyon shape. Compared to MSP, the localized deep posterior staphyloma was a well-defined dark-brown round excavated area with pigmentation in the superior part. In the eyes of the localized deep posterior staphyloma, fluorescein and indocyanine green angiography showed cilioretinal artery branching nasally, dipping in and emerging from the temporal margin. OCT showed thinning and outpouching of outer retina, RPE, choroid, and sclera with intact inner retina [10]. The phenotype of a scleral pit seems a plausible rationale, but its correlation with the pathogenesis and histological morphology of posterior scleral staphyloma is yet to be investigated.

In this study, we retrospectively analyzed eight cases of PM with MSP and integrated them with the other two reported cases. Both visual ability and the size of MSPs were relatively stable in the patients who were followed up for more than 6 months, of whom the longest follow-up was more than three years. We also found that there was no correlation between logMAR BCVA and the size of pits. The reason for this may be that the pit was less extensive and there was still some distance from the macula lutea. Therefore, it was likely that other severe ocular complications contributed to visual impairment. The evidence stated that MSP is different from other diseases, such as focal choroidal excavation, optic disc pit, macular coloboma, and posterior scleral staphyloma. Such damage is rare and suggests that the scleral curvature of the macular chorioretinal atrophy area in PM eyes might alter significantly. Thus, we concluded that MSP might be a scarce physical sign of PM or congenital anomalies of the sclera. Due to the limited number of cases and follow-up time, additional in-depth studies are required to explain the histomorphological characteristics, disease progression, and impact on patients’ vision.

## Data Availability

All data generated or analysed during this study are included in this article. Further enquiries can be directed to the corresponding author.
